# Seasonal dynamics in bacterial communities of closed-cage broiler houses

**DOI:** 10.3389/fvets.2022.1019005

**Published:** 2022-11-07

**Authors:** Huan Chen, Han Yan, Yan Xiu, Linlin Jiang, Jianlong Zhang, Guozhong Chen, Xin Yu, Hongwei Zhu, Xiaoyu Zhao, Youzhi Li, Wenli Tang, Xingxiao Zhang

**Affiliations:** ^1^College of Life Science, Ludong University, Yantai, Shandong, China; ^2^Shandong Breeding Environmental Control Engineering Laboratory, Ludong University, Yantai, China; ^3^Clinical Lab, Yantai Affiliated Hospital of Binzhou Medical University, Yantai, China; ^4^Yantai Key Laboratory of Animal Pathogenetic Microbiology and Immunology, Ludong University, Yantai, China; ^5^Shandong Provincial Key Laboratory of Quality Safety Monitoring and Risk Assessment for Animal Products, Institute of Veterinary Drug Quality Inspection of Shandong Province, Jinan, China

**Keywords:** broiler house, 16S rDNA, high-throughput sequencing, bacteria, aerosol

## Abstract

The bacteria contained in air aerosols from poultry houses are closely connected to animal health and production. This study aimed to investigate the seasonal factors on microbial aerosol concentration, particle size and bacterial spectrum composition inside a closed-cage broiler house. Then, 16S rDNA sequencing technology was applied to analyze the characteristics of bacterial abundance and diversity. The results indicated that the concentration of bacterial aerosol in the broiler house varied significantly in different seasons, with a concentration range of 5.87–15.77 × 10^3^ CFU/m^3^, and the highest and lowest concentrations in the summer and winter, respectively. Microbiological analysis showed that the proportion of Gram-negative bacteria in autumn was significantly higher than that in summer (*P* < 0.05). In addition, the floral structure of potential pathogenic bacterial genera also differed by season. *Escherichia-Shigella, Streptococcus, Acinetobacter, Pseudomonas* were identified in the bacterial aerosols. Importantly, the relative abundance of *Firmicutes* in spring and autumn was much higher. In contrast, the relative abundance of *Proteobacteria* in spring and autumn was lower than that in summer and winter. Altogether, results revealed the effects of seasonal factors on the diversity and abundance of bacteria and the distribution characteristics of major opportunistic pathogens in the air of closed-cage broiler houses. These results will provide important information for exploring the potential risk of aerosols from poultry houses all four seasons.

## Introduction

Modern poultry farming in intensive buildings with high animal densities can lead to poor indoor air quality. The aerosols formed by microorganisms and their metabolites in the environment are emitted into the air. This outcome not only pollutes the environment but also affects the health of animals and farmers (1–3). Furthermore, such aerosols can lead to a prevalence of respiratory diseases that can cause declines in animal production. Studying the diversity and community structure of airborne microorganisms in livestock houses is conducive to understanding the impact of bioaerosols on the health of employees and animals (4, 5).

To better understand the microbial species and quantities in chicken houses exposure to contaminated environments, the characteristics of the bioaerosol components have been investigated using different approaches. Molecular biological techniques were used for microbial research, including fluorescence quantification, bacterial tag-encoded flexible amplicon pyrosequencing (6), and denaturing gradient gel electrophoresis (7), among others (8). These methods can only detect partial microorganisms or specific pathogenic microorganisms, and they are very limited in their ability to determine species and quantity. In recent years, the development of molecular detection methods, especially the emergence of high-throughput sequencing and metagenomics technologies, provided an effective means to overcome these shortcomings (9, 10). High-throughput sequencing technology has been used to analyze bioaerosols in livestock houses (11, 12). Different community structures may lead to differences in the effects of bioaerosols on livestock health (13). Although scholars have started to study the community structure of bioaerosols in livestock houses, there are still many issues to be clarified.

During livestock and poultry farming, the structure of the bacterial aerosol inside the chicken house is affected by many factors. Because of the influence caused by the types of animal houses, feeding methods, environmental factors and so on, the microbial community structure and quantities in chicken houses have significant variations. Multiple studies have shown that concentrations of airborne microbes in chicken houses often exceed 10^6^ CFU/m^3^ (14–16). In spring, the cage broiler houses have a relatively high abundance of pathogenic bacteria including *Acinetobacter, Pseudomonas, Enterococcus, Microbacterium* which were identified by the high-throughput 16S rDNA sequencing technique (12). Another study explored that *Faecalibacterium, Escherichia* and *Corynebacterium* were the dominant microbes in layer caged houses in summer (11). To date, only a few studies have been performed to evaluate the effects of seasonal factors on the quantity of microorganisms and their distribution in the air environment in broiler houses. Although several pathogenic bacteria have been observed in previous reports, how the seasons affect the distribution of opportunistic pathogens, including those that cause respiratory allergies and infections, as well as other pathogenic bacteria, remain unknown (17, 18).

The purpose of this study was to investigate the size, structure, and distribution of the microbial particles in a caged broiler house during different seasons. The study also objectively evaluated the environmental status of a broiler house under the cage mode to provide theoretical approaches for environmental regulation and disease prevention, as well as for controlling intensive broiler production.

## Materials and methods

Procedures involving animals complied with the Animal Research Ethics Committee of Ludong University. A trial was conducted at the Muping Farm of The Xiantan Company Limited of Yantai, in Shandong Province, China.

## Selection of sampling location

The sampling location was an intensive closed-cage broiler house in the Muping District, Yantai City, Shandong Province, China. The broilers were raised in the middle floor of a 3 tier set of overlap cages. Individual cages were 70 cm in length × 40 cm in width × 38 cm in height, and contained two nipple drinkers. Excreta was collected in trays under each cage, and was removed every 2 or 3 days to decrease ammonia concentrations in the broiler house. The sampling seasons (months) were spring (April), summer (August), autumn (October) and winter (December). The chicken house was aligned north to south. The house was 100 m long, 14 m wide, 3.2 m high, and it contained approximately 18,000 broilers. The in-house temperature in spring was 20–22°C, in summer was 26–28°C, in autumn was 20–22°C, and in winter was 16–18°C. The humidity was 55–65%. The equipment in the house was complete, including fans and wet curtain, longitudinal mechanical ventilation, fully automatic feed and water lines, and free access to food and drinking water. The points of detection included the front, middle, and rear.

## Sample collection

Culturable airborne bacteria were collected using the international standard Andersen 6-staged airborne microbe sample collector (JWL6, Beijing, China). This device has 6 stages with 400 holes per stage. From top and bottom, the pore size gradually decreases, and bioaerosol particles are captured on Petri dishes placed in each stage of the sampler based on their size (19). Blood agar medium was used for sampling, the sample collection time was 2 min, and the gas flow rate was 28.3 L/min. The flow rate of the sampling pump was calibrated with a flow meter before each sampling. All the sample collector was performed at a height of 0.5 m above the ground (the height of the chicken's nasal cavity). After sampling, the blood agar medium was aerobically cultured in an incubator at 37°C for 48 h. The total number of aerobic bacteria was recorded. The number of colonies was corrected *via* the positive hole method. According to the sampling time and gas flow rate, the number of airborne bacteria-bearing particles at each stage was calculated using the following formula: *C* = (*N* × 1,000)/(*t* × *F*), where C is the bacterial aerosol concentration, colony-forming unit (CFU)/m^3^; *N* is the number of colonies at each stage; *t* is the sampling time, min; and *F* is the gas flow rate at the time of sampling, L/min. The samples were used for the particle size distribution of the airborne bacteria.

The total bacterial aerosol was collected using a BioSampler sampler (SKC, USA) with a gas flow rate of 12.5 L/min, a sampling time of 1 hour, and a sampling height of 0.5 m. The collected liquid containing airborne microorganisms was ultracentrifuged and stored at −80°C. The samples were used for microbiota analysis.

## 16S rDNA analysis

The EasyPure Viral DNA/RNA Kit (Transgen Biotech, China) was used to extract RNA from the concentrate containing the airborne microorganisms, and the cDNA was synthesized *via* reverse transcription using the cDNA Synthesis SuperMix Kit (Transgen Biotech, China). The DNA samples were analyzed by Novogene Biotech Co., Ltd, Beijing, China, *via* high-throughput sequencing of the V4–V5 region of the bacterial 16S rDNA gene using the Illumina HiSeq 2,500 sequencing platform (20, 21). The data were deposited with links to BioProject accession number PRJNA784692 in the NCBI BioProject database.

## High-throughput sequencing and sequence analysis

Barcoded 515F/907R primers were used to PCR amplify the V4–V5 region of the 16S rDNA gene and barcoded amplicons were pooled with equal concentrations. The resulting sequencing reads were firstly separated according to barcode and primer sequences using custom Perl scripts. The sequencing reads were paired-end joined, filtered based on the quality score using QIIME software, and culled out the sequences with chimeras detected by UCHIME. Remained sequences were clustered into operational taxonomic units at 97% similarity. Resulting operational taxonomic units (OTUs) were taxonomically classified by Ribosomal Database Project (RDP) Naïve Bayesian Classifier with training set.

Based on the OTUs generated *via* sample sequencing, the alpha diversity of each sample was calculated. This approach gives the species diversity of the individual samples, including the Chao1 value, the ACE value, and the Shannon index. The Chao1 and ACE values predict the species of microorganisms (the number of OTUs) in the sample based on the number of obtained tags and the OTUs. The Shannon index reflects diversity: high Shannon index values indicate a large abundance of a species in the sample. Beta diversity analysis was performed using UniFrac distance metrics and visualized *via* principal coordinate analysis (PCoA). The weighted UniFrac distance reflects the difference in abundant lineages. Subsequently, the samples were subjected to clustering analysis to determine the relative abundance of the species compositions of each sample. The similarity and variability of total bacteria and pathogenic bacteria were compared among different samples (22, 23).

## Data statistics and analysis

SPSS statistical software (version 19.0; SPSS Inc., USA) was used. One-way analysis of variance (ANOVA) was performed between different groups. And Turkey HSD test was applied for *post-hoc* comparisons at α = 0.05 as needed. The experimental data are expressed as the mean ± standard deviation. *P* < 0.05 represents a significant difference, and *P* < 0.01 represents an extremely significant difference. The data were graphed using GraphPad Prism 7 (GraphPad Software, California, USA).

## Results

### Bacterial concentrations in the broiler house

**Figure 1:**
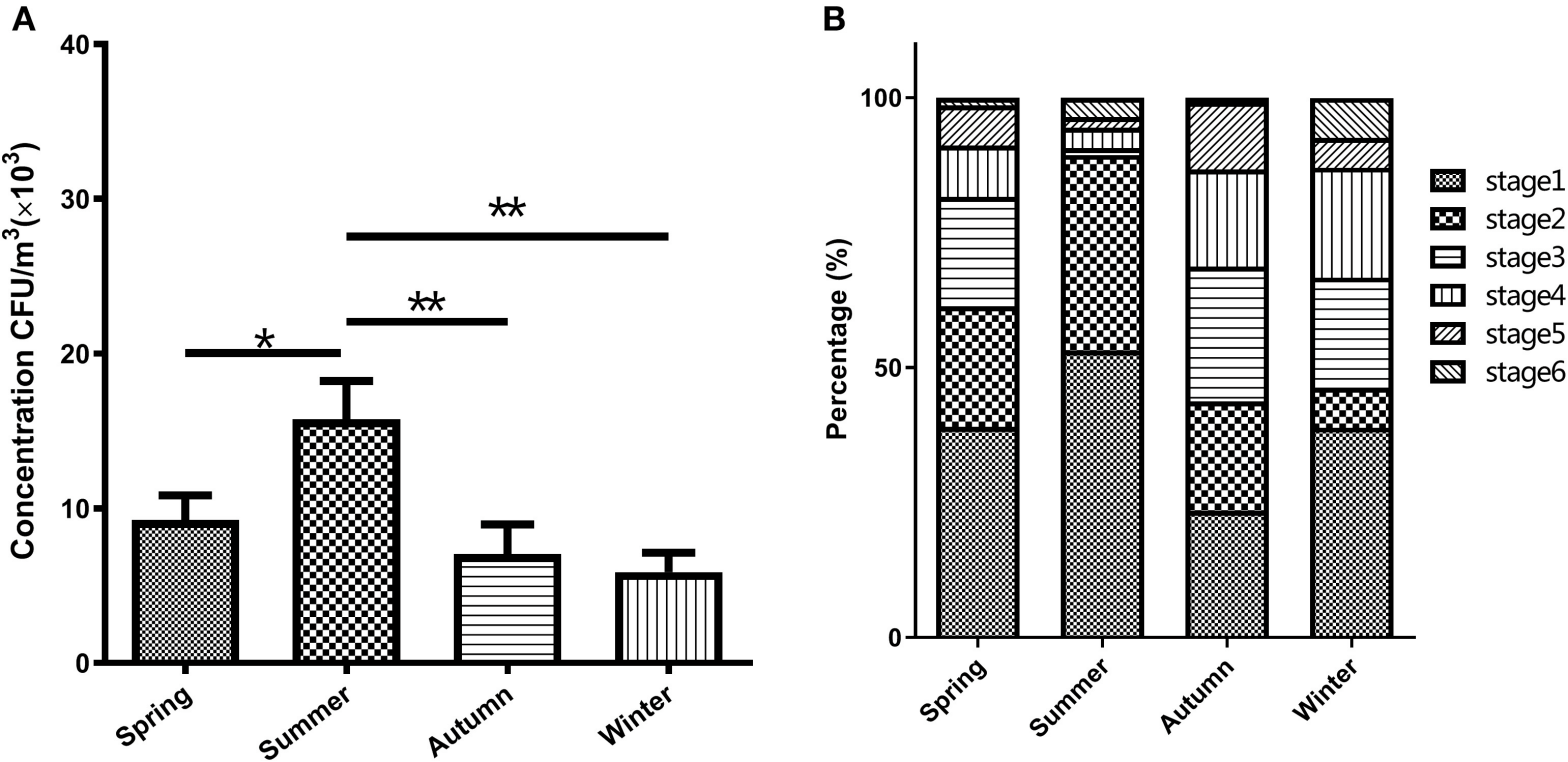
Characteristics of the airborne bacterial aerosol concentration in the broiler house **(A)** and size distribution of airborne cultivable- bacterium-containing particles in the broiler house **(B)**. Size-resolved airborne cultivable bacterial levels were obtained with the Andersen six-stage cascade impactor, expressed as percentages of the total level and categorized into four groups: Spring, Summer, Autumn, and Winter. Aerodynamic diameter ranges for the viable particle sizing sampler were >7.0 μm (stage 1), 4.7–7.0 μm (stage 2), 3.3–4.7 μm (stage 3), 2.1–3.3 μm (stage 4), 1.1–2.1 μm (stage 5), and 0.6–1.1 μm (stage 6). The bars in **(A)** show the means ± SD (*n* = 3). *P < 0.05; **P < 0.01.

The bacterial concentrations in the broiler house during different seasons ranged from 5.87 × 10^3^ to 15.77 × 10^3^ CFU/m^3^ (Figure 1A). The one-way ANOVA demonstrated that the total bacterial concentration in four seasons had a significant different result (*p* < 0.001). Specifically, according to the *post hoc* test, the bacterial concentration in summer showed significantly higher than that in spring (*p* < 0.05), autumn (*p* < 0.01), and winter (*p* < 0.01). As shown in Figure 1A, the total concentration of airborne bacteria was highest in the summer, about two times the total bacterial concentration measured during the winter.

### Characteristics of the bacterial aerosol in various seasons

As shown in Figure 1B, the particle size distribution of the airborne bacteria varied seasonally. The bacterial particles were mainly distributed in the first 4 stages (>2.1 μm), which accounted for 91.16 and 94.43% of the totals in spring and summer, respectively, and 86.72 and 87.04% of the totals in autumn and winter, respectively. The peaks were distributed at stage 1 (>7.0 μm), which accounted for 39.07, 53.18, and 38.89% in spring, summer, and winter, respectively. The peak during autumn was distributed at stage 3 (3.3–4.7 μm), which accounted for 25.00%. The lowest values during spring and autumn were distributed at stage 6 (< 1.1 μm), accounting for 1.40 and 0.78%, respectively. The lowest values during summer and winter were distributed at stage 5 (1.1–2.1 μm), accounting for 1.99 and 5.56%, respectively. In autumn, the fraction of the bacterial particles capable of entering the lungs (stages 3–6) was the highest, accounting for 56.25% of the total number of particles, while the fraction of particles in this size range was the smallest in the summer, accounting for 10.61% of the total number of particles.

### Microbiome sequencing data analysis

The total number of raw sequences in the samples was 966,428. After filtering the low-quality sequences, the effective tags obtained were arranged from 78,431 to 32,988. Sequences with similarities greater than 97% were assigned to the same OTU. The average number of OTUs per sample was 431 ± 45.24, ranging from 259 to 839 OTUs.

**Figure 2:**
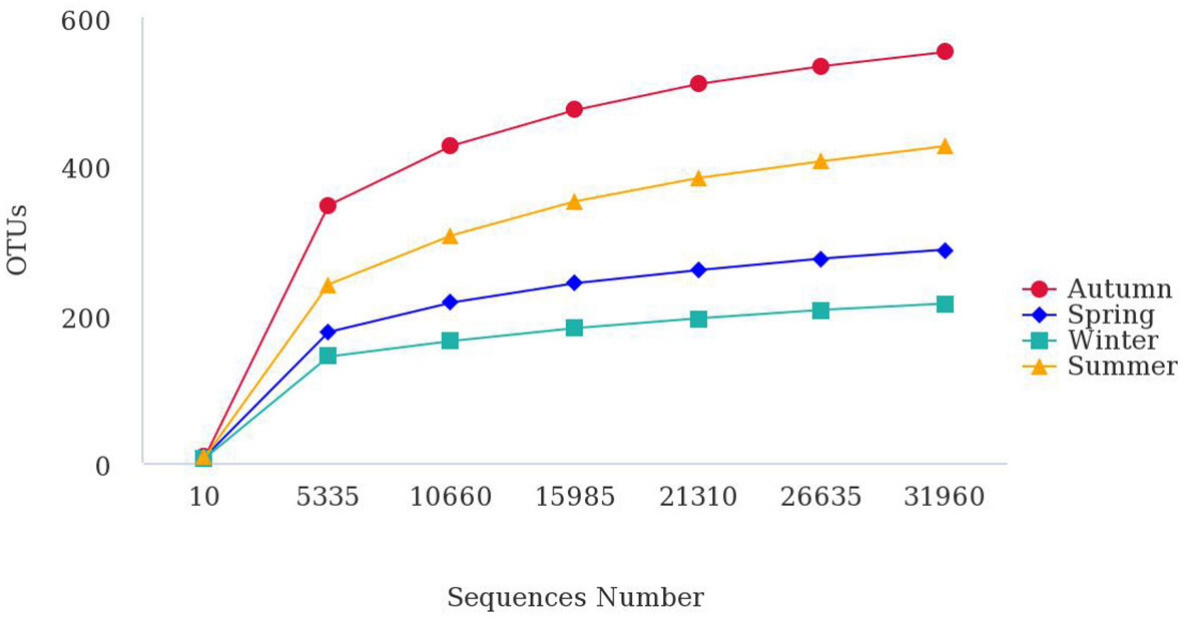
Rarefaction curves describe the bacterial OTU richness in the bioaerosol during different seasons. Diversity indices were calculated using random selections of 31,960 sequences per sample.

To compare diversity levels and satisfactory coverage, samples were compared with rarefaction analysis (Figure 2). Almost all bacteria in the samples were covered when the number of sequencing samples exceeded 30,000 reads, indicating that the 16S rDNA sequences represented the microbial diversity of the samples. At this depth of coverage, bacterial richness was significantly higher in autumn in comparison to winter.

### Analysis of bacterial community composition

**Figure 3:**
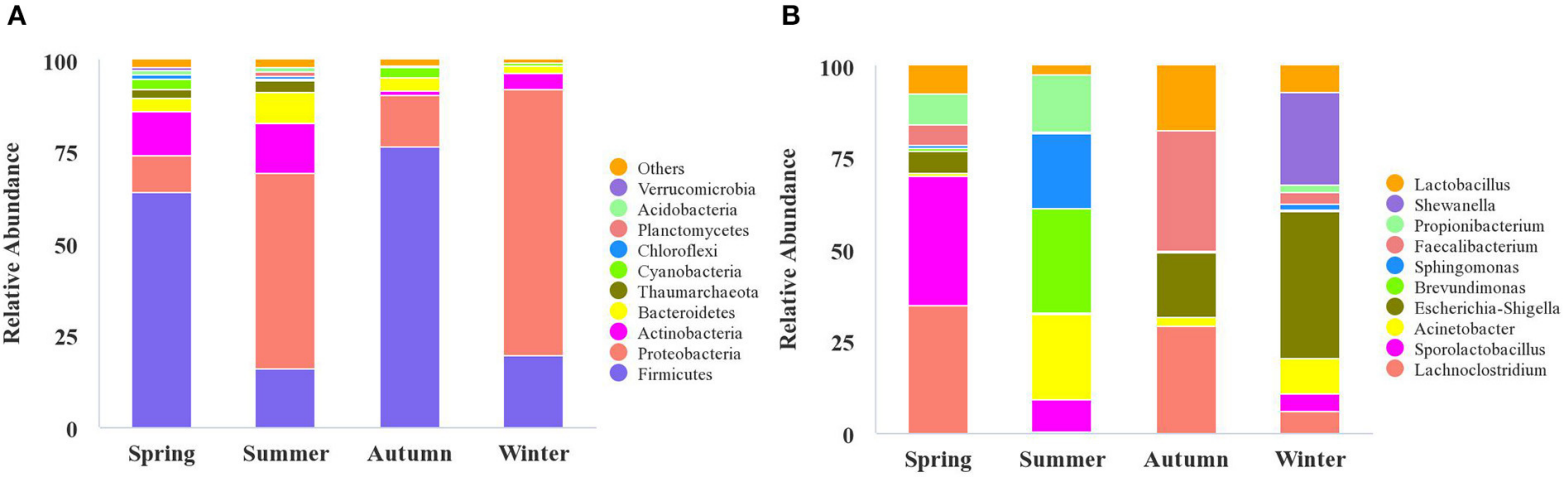
The microbiota composition in the air of broiler house. The relative abundance of OTUs was used to determine the bacterial composition at phylum **(A)** and genus **(B)** levels. In A and B, the abscissa shows the sample names, the ordinate shows the proportions of the species in the samples, the columns of different colors represent different species, and the lengths of the columns represent the proportions of the species. Only the top 10 phyla and genera are displayed, with unidentified and lowly abundant bacteria collectively denoted as “others.”

**Figure 4:**
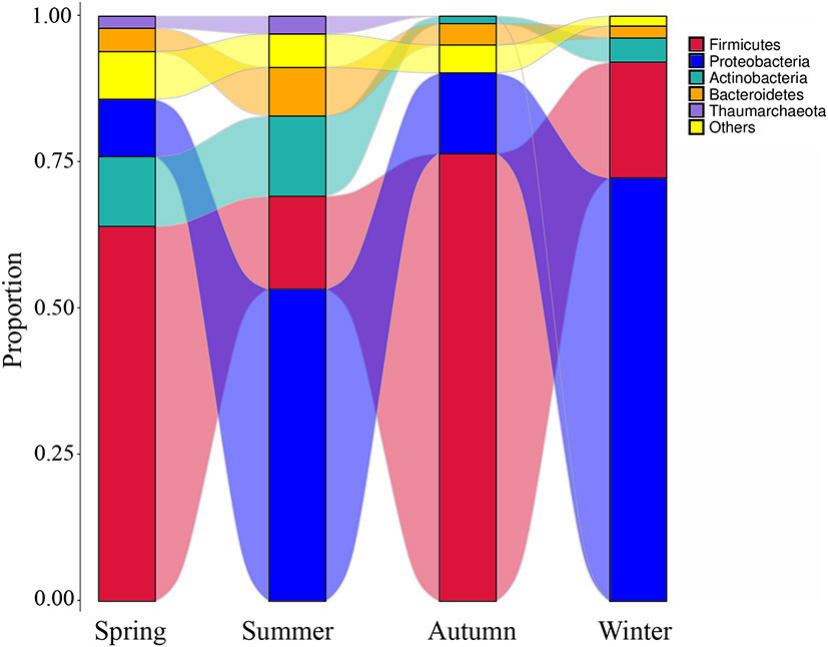
The microbial composition of the air of broiler house in different seasons. The Sankey Diagram was constructed with the relative abundance of OTUs to determine the bacteria composition of different seasons in the broiler house.

High-throughput sequencing identified 39 phyla, 261 families, and 521 genera of bacteria, indicating that the airborne microbes in the broiler house are diverse and abundant. In order to analyze samples of different seasons, the 10 most frequent phyla were listed in descending order starting with the most statistically significant: *Firmicutes, Proteobacteria, Actinobacteria, Bacteroidetes, Thaumarchaeota, Cyanobacteria, Chloroflexi, Planctomycetes, Acidobacteria, Verrucomicrobia* (Figure 3A). The graph shows that the *Firmicutes* abundance was high in autumn (76.48%), and the *Proteobacteria* abundance was high in winter (72.36%). The most abundance bacterium in summer was *Bacteroidetes*, accounting for 8.32%. Tracking individual OTUs within different phyla revealed distinct temporal dynamics within the *Firmicutes* and *Proteobacteria* by the Sankey diagram (Figure 4). Interestingly, the relative abundance of *Firmicutes* in spring and autumn was much higher than that in summer and winter. In contrast, the relative abundance of *Proteobacteria* in spring and autumn was lower than that in summer and winter.

**Figure 5:**
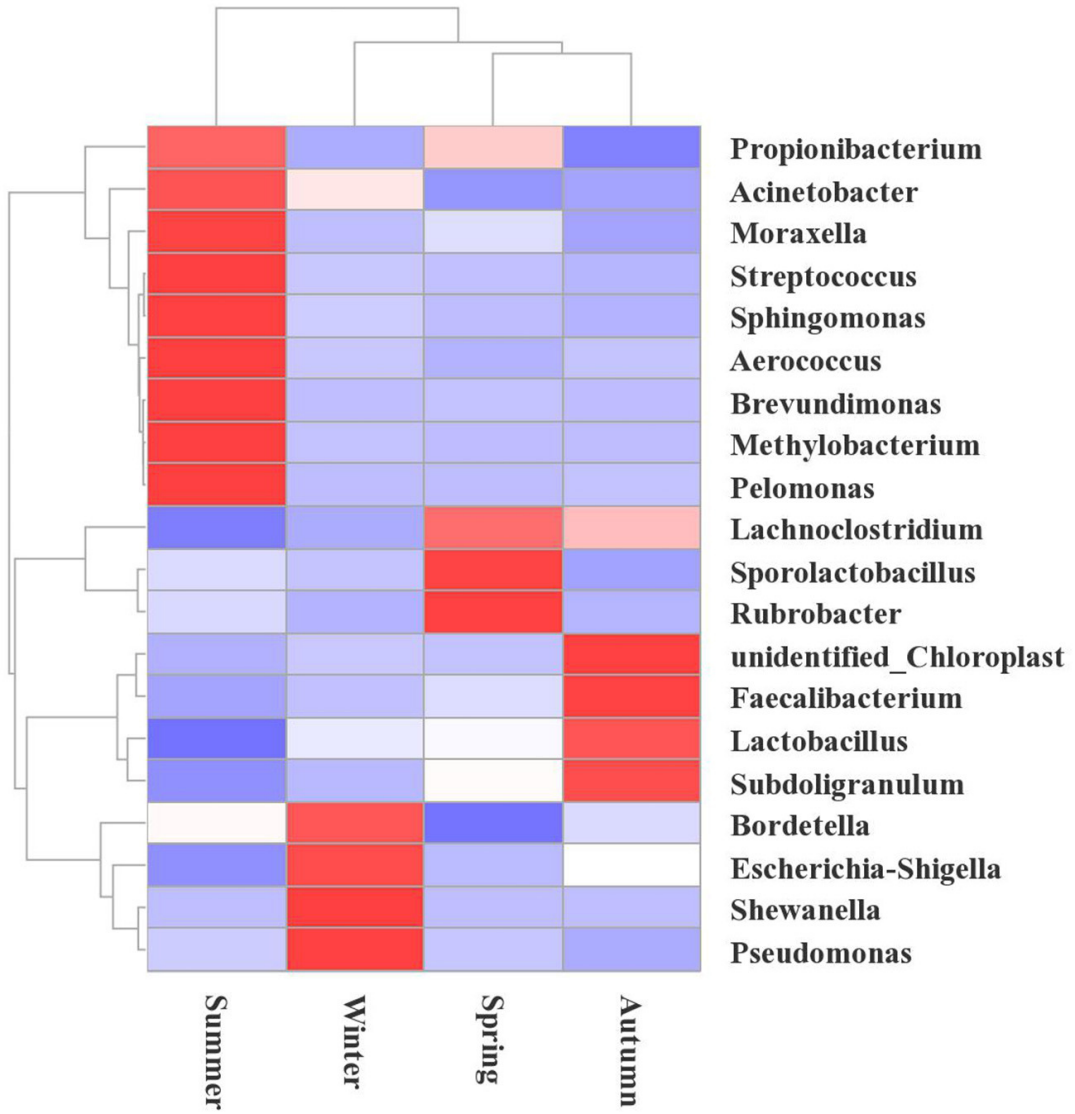
Heatmap showing the changes in the microbial communities at the genus level during the different seasons in the broiler house. The species cluster tree is shown on the left, and the sample cluster tree is shown on the top. The colors reflect the similarities and differences of the highly rich bacterial community compositions in the broiler house during different seasons. The red gradient indicates higher community richness, and the blue gradient color indicates lower community richness.

The relative abundance of bacteria from the 10 most frequent genera differed depending on the seasonal factors. These relations are illustrated using the column diagram (Figure 3B), and the heatmap (Figure 5). *Shewanella* was the dominant flora in winter, accounting for 9.01%, while it was not detected during the other seasons. In the spring, the airborne *Lachnoclostridium* abundance was higher than during the other seasons, accounting for 12.54%. The abundances of the opportunistic pathogens *Escherichia-Shigella* and *Pseudomonas* were highest in winter, accounting for 14.23 and 4.00%, respectively (Figure 5).

According to a hierarchical cluster analysis, the summer bacterial community differed significantly from those in the other samples (Figure 5). The predominant bacteria were *Brevundimonas, Acinetobater, Sphingomonas, Methylobacterium*, and *Propionibacterium*. The floral structures in the spring and autumn were similar, as they clustered tightly, and the dominant floras were *Faecalibacterium, Lachnoclostridium*, and *Escherichia-Shigella*.

**Figure 6:**
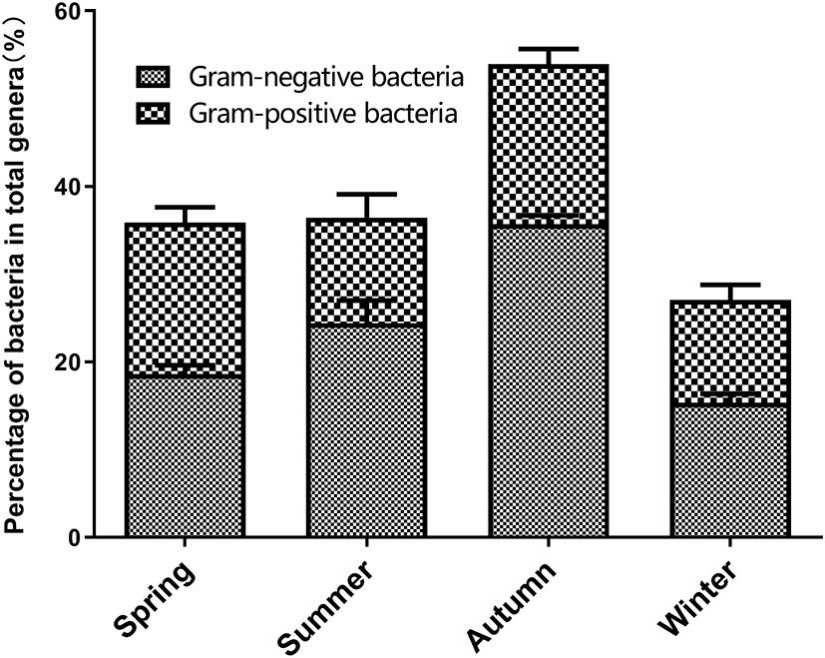
The percentage of gram-negative bacteria and gram-positive bacteria for total genera in four seasons.

The varieties analysis of Gram-negative and Gram-positive bacteria during the different seasons is shown in Figure 6. During the different seasons, the strains of Gram-negative bacteria was significantly higher than that of Gram-positive bacteria. In autumn, the numbers of Gram-positive and -negative bacterial genera in the broiler house were significantly higher than their numbers during the other seasons (*P* < 0.05). In summer, the number of Gram-positive bacterial genera was lower than spring and autumn. The animal pathogens with relatively high abundances (>0.1%) in the air samples from each season were dissected. Table 1 shows the distribution characteristics of 12 potential opportunistic pathogens detected in air samples from the broiler house. These 12 opportunistic pathogens belong to the phyla *Firmicutes, Proteobacteria, Bacteroidetes*, and *Actinobacteria*.

**Table 1:** Distribution characteristics of potential animal pathogens in air samples from the broiler house.

**Genus**	**Phylum**	**Percent (%)**			
		**Spring**	**Summer**	**Autumn**	**Winter**
*Acinebobacter* (24)	*Proteobacteria*	0.29	7.02	0.65	3.43
*Bacillus* (25)	*Firmicutes*	0.18	0.26	0.50	0.13
*Bacteroides* (26)	*Bacteroidetes*	0.31	0.00	0.94	0.35
*Brevundimonas* (27)	*Proteobacteria*	0.24	8.55	0.04	0.11
*Clostridium* (28)	*Firmicutes*	0.02	0.67	0.02	0.00
*Corynebacterium* (29)	*Actinobacteria*	0.20	3.81	0.09	0.41
*Enterococcus* (30)	*Firmicutes*	0.24	0.00	1.08	0.31
*Prevotella* (31)	*Bacteroidetes*	0.27	0.00	0.00	0.00
*Pseudomonas* (32)	*Proteobacteria*	0.50	0.57	0.13	4.00
*Staphylococcus* (33)	*Firmicutes*	0.77	2.78	0.36	0.51
*Stenotrophomonas* (34)	*Proteobacteria*	0.06	0.37	0.10	0.11
*Streptococcus* (35)	*Firmicutes*	0.11	2.92	0.00	0.20

### Beta diversity in the bacterial community

**Figure 7:**
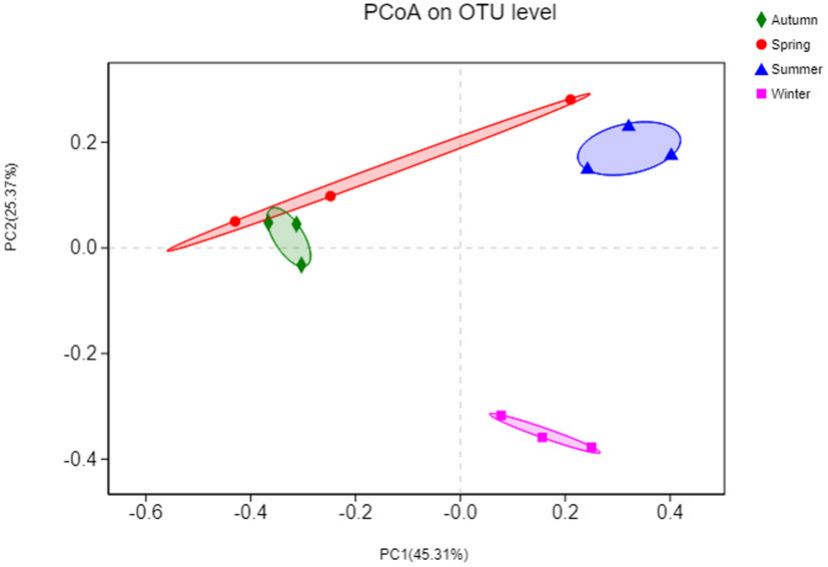
Principal coordinates analysis (PCoA) based on the numbers of OTUs in the bacterial communities. The x-axis shows the first principal component and the y-axis shows the second principal component. Data points shown with different colors represent groups of samples under different conditions.

Initially, the differences in OTU composition were examined *via* principal coordinate analysis (PCoA) (Figure 7). Phylogenetic beta diversity analyses were then performed on those replicates based on the weighted UniFrac distances. The bacterial compositions of different samples were more related to season because the samples could be clustered based on the different seasons. The PCoA revealed that the OTU composition in summer was clearly distinct from those in other three seasons (*P* < 0.05). In the PCoA chart, the spring and autumn samples partially overlapped, suggesting that there are variations in the beta diversity of the floral structures in each seasons.

## Discussion

### Differences in bacterial aerosol concentration in the broiler house

The number of airborne microorganisms in livestock houses is an important indicator of the environmental quality. The bacteria concentrations in previous poultry studies were range from 4.6 × 10^5^ to 4.2 × 10^10^ CFU/m^3^ bacteria (36, 37). This study revealed significant seasonal variations in the content of the bacterial aerosol in a broiler house, with a concentration range of 5.87 × 10^3^-15.77 × 10^3^ CFU/m^3^ over all four seasons. In this study, the entire range of bacterial concentrations in four seasons exceeds the safety limit for the recommended bacterial concentration in the indoor air quality standard proposed by the American Conference of Governmental Industrial Hygienists (ACGIH) (500 CFU/m^3^) (38). While most bacterial concentrations are still under the broiler house air microbial content standard (2.5 × 10^4^ CFU/m^3^) specified in China's “Environmental Quality Standard for Livestock and Poultry Farm” NY/T 388-1999. Furthermore, from the perspective of the distribution characteristics, the microbial concentration in the broiler house was characterized by a high level in summer and a low level in winter. The reason for this observation is that the temperature and humidity are high during summer, which are beneficial to the growth of microorganisms. This result was consistent with Jo's research (39), which showed that the bacterial concentration in summer was higher than that in winter. The bacterial aerosol concentrations in spring and autumn were relatively similar, which might be because these seasons have similar air temperatures, and temperature is a key factor that affects the bioaerosol concentration in the house.

The particle size distribution of a bioaerosol directly determines the sedimentation location of the particles in the respiratory system after inhalation by animals. Particles larger than 7 μm (equivalent to stage 1 of the 6-staged sampler) are trapped in the nasal cavity, and particles of 4.7–7 μm (equivalent to stage 2 of the 6-staged sampler) can reach the bronchus. Bioaerosols with a diameter smaller than 4.7 μm (equivalent to stages 3–6 of the 6-staged sampler) might easily penetrate the respiratory system. The results of this study showed that the aerosol concentration in the range of 0.65–2.1 μm was higher in winter than in other the seasons, accounting for 7.41%. This fraction of the bacterial aerosol has a small particle size and is prone to settle to the lungs. During the other three seasons, the maximum values were all distributed at >7.0 μm, and this fraction of particles has fewer effects on animals. This analysis of the particle size distribution characteristics will provide basic data for the assessment of health risks and environmental pollution with bacterial aerosols during broiler cultivation.

### The structure and biodiversity of the broiler house bacterial community

Traditional culture methods can collect and culture less than 10% of airborne microorganisms, and the number of microorganisms changes during the medium collection process (40). Therefore, these methods are not useful for studying the diversity and richness of airborne microorganisms. The prokaryotic 16S rDNA gene sequence is highly conserved but has some variability. Sequencing the V4–V5 region of 16S rDNA with the Illumina Hiseq sequencing technology successfully detected seasonal variations in the diversity in the airborne bacterial community structure in the broiler house, and comprehensive and in-depth microbial community information was obtained.

High-throughput sequencing identified 39 phyla, 261 families, and 521 genera of bacteria, indicating that the airborne microbes in the broiler house are diverse and abundant. *Firmicutes, Proteobacteria, Actinobacteria*, and *Bacterioidetes* were the dominant bacterial phyla during all four seasons, while the bacterial community structure in the air of the broiler house varied by season. *Firmicutes* were highly abundant during spring and autumn. These findings were consistent with those obtained when studying air in poultry-confinement buildings (41). The genus *Enterococcus*, which belongs to *Firmicutes*, was significantly more abundant during autumn (1.08%) than during spring (0.24%), summer (0.00%), and winter (0.31%). *Proteobacteria* is the largest phylum of bacteria, and it includes many opportunistic pathogens. The *Proteobacteria* abundance was significantly higher during winter (72.36%) than during spring (9.82%), summer (53.37%), and autumn (13.82%). For example, the abundance of the genus *Pseudomonas* (which belongs to the phylum *Proteobacteria* and is a conditional pathogen), was significantly higher in winter (4.00%) than in spring (0.50%), summer (0.57%), and autumn (0.13%). *Acinetobacter* is ubiquitous in natural environment (42, 43). *Acinetobacter* is a Gram-negative pathogen, which triggers infection in different part of the human body, such as lung infection and pneumonia (44). *Acinetobacter* was found in air samples of the four seasons in this study. In our study, the kinds of Gram-negative bacteria were more than that of Gram-positive bacteria. These results correspond to research by others (15, 45).

### The relative abundance of bacterial communities in the broiler house

The dominant airborne genera in the broiler house during the different seasons were *Lachnoclostridium, Escherichia-Shigella, Lactobacillus*, and *Faecalibacterium*. High-throughput sequencing results showed that the proportions of Gram-negative bacteria in the autumn were significantly higher than those in summer. For example, *Faecalibacterium* accounted for 10.10% in autumn, higher than in other seasons (2.12% in spring, 0.08% in summer, and 1.09% in winter). The dominant airborne genera in this study did not exactly correspond to previous poultry studies (13, 46), probably because the dominant bacteria depend on seasonal factor, such as the control of relative temperature and humidity. Because relatively few research about seasonal bioaerosol diversity in poultry houses in China, all of these factors could have potentially contributed to the different dominant bacterial genera reported here.

A large number of opportunistic pathogens were detected in the bacterial aerosol. An analysis of the potential animal pathogens in the air samples identified six species of opportunistic pathogens that were present during all four seasons, and another six animal pathogens that were only detected in some of the air samples. For example, *Prevotella* was only present in spring samples and was not detected during the other three seasons. The analysis showed that the number of *Enterococcus* and *Pseudomonas* in the broiler house was the highest in winter, which is likely due to poor ventilation in the winter house. Such analyses of potential animal pathogens have important clinical implications. For example, pathogenic *Staphylococci* often cause staphylococcal disease in chickens (47, 48). *Pseudomonas* can lead to most of the sick chickens demonstrating acute sepsis, with high morbidity and mortality (49, 50). Considering the conditional pathogen bacterial diversity, rather than total the quantity of bioaerosols, may better explain that the broiler health can be affected by the seasonal factor (7). The results of the study proved that the dynamic patterns of microbial aerosols in closed cage broiler houses at different seasons.It is important for establishing risk assessment systems and managing the pathogen aerosols present during livestock and poultry farming. This study provides a foundation for assessing the health risk of biological aerosols in the atmosphere of intensive poultry houses.

## Conclusion

In this study, the results showed that the relative abundances of the dominant genera varied during the different seasons in the cage mode. In summer, although the total bacterial aerosol concentration was high, the bacterial diversity was low. In autumn, the bacterial community structure was highly diverse. The bacterial aerosol concentration was low in winter, while the numbers of genera and opportunistic pathogens kept at high levels. This study is of practical significance, as the results could be useful for improving the air environment in broiler houses and for guiding broiler production.

## Data availability statement

The datasets presented in this study can be found in online repositories. The names of the repository/repositories and accession number(s) can be found in the article/Supplementary material.

## Author contributions

HC and LJ designed the experiments. HY, YX, GC, and XY carried out the experiments. JZ, XZhao, and WT analyzed the experimental results. HZ and YL wrote the manuscript. XZhan reviewed and edited the manuscript. All authors contributed to the article and approved the submitted version.

## Funding

This work was supported by the Natural Science Foundation of Shandong Province (ZR2020KC028 and ZR2020QC227), Innovation Team Project for Modern Agricultural Industrious Technology System of Shandong Province (SDAIT-11-10), and Key Research and Development Plan of Yantai (2021XDHZ076 and 2021XDHZ084).

## Conflict of interest

The authors declare that the research was conducted in the absence of any commercial or financial relationships that could be construed as a potential conflict of interest.

## Publisher's note

All claims expressed in this article are solely those of the authors and do not necessarily represent those of their affiliated organizations, or those of the publisher, the editors and the reviewers. Any product that may be evaluated in this article, or claim that may be made by its manufacturer, is not guaranteed or endorsed by the publisher.
