# Supplementary material for: Seasonal dynamics in bacterial communities of closed-cage broiler houses
Source: Front Vet Sci. 2022 Nov 7;9:1019005. doi: 10.3389/fvets.2022.1019005 (PMC9669973; doi:10.3389/fvets.2022.1019005)
Supplement: Supplementary file 1 [file Table_1.DOCX]

Table 1. Distribution characteristics of potential animal pathogens in air samples from the broiler house.

| Genus | Phylum | Percent (%) | | | |
| --- | --- | --- | --- | --- | --- |
|  |  | Spring | Summer | Autumn | Winter |
| *Acinebobacter* (McConnell et al., 2013) | *Proteobacteria* | 0.29 | 7.02 | 0.65 | 3.43 |
| *Bacillus* (Ehling-Schulz et al., 2019) | *Firmicutes* | 0.18 | 0.26 | 0.50 | 0.13 |
| *Bacteroides* (Falagas and Siakavellas, 2000) | *Bacteroidetes* | 0.31 | 0.00 | 0.94 | 0.35 |
| *Brevundimonas* (Liu et al., 2021) | *Proteobacteria* | 0.24 | 8.55 | 0.04 | 0.11 |
| *Clostridium* (Mehdizadeh Gohari et al., 2021) | *Firmicutes* | 0.02 | 0.67 | 0.02 | 0.00 |
| *Corynebacterium* (Rajamani Sekar et al., 2017) | *Actinobacteria* | 0.20 | 3.81 | 0.09 | 0.41 |
| *Enterococcus* (Fiore et al., 2019) | *Firmicutes* | 0.24 | 0.00 | 1.08 | 0.31 |
| *Prevotella* (Larsen, 2017) | *Bacteroidetes* | 0.27 | 0.00 | 0.00 | 0.00 |
| *Pseudomonas* (Silby et al., 2011) | *Proteobacteria* | 0.50 | 0.57 | 0.13 | 4.00 |
| *Staphylococcus* (Cheung et al., 2021) | *Firmicutes* | 0.77 | 2.78 | 0.36 | 0.51 |
| *Stenotrophomonas* (Brooke, 2012) | *Proteobacteria* | 0.06 | 0.37 | 0.10 | 0.11 |
| *Streptococcus* (Kadioglu et al., 2008) | *Firmicutes* | 0.11 | 2.92 | 0.00 | 0.20 |
