# Supplementary material for: Seasonal dynamics in bacterial communities of closed-cage broiler houses
Source: Front Vet Sci. 2022 Nov 7;9:1019005. doi: 10.3389/fvets.2022.1019005 (PMC9669973; doi:10.3389/fvets.2022.1019005)
Supplement: Supplementary file 2 [file Presentation_1.PPTX]

## Slide 1
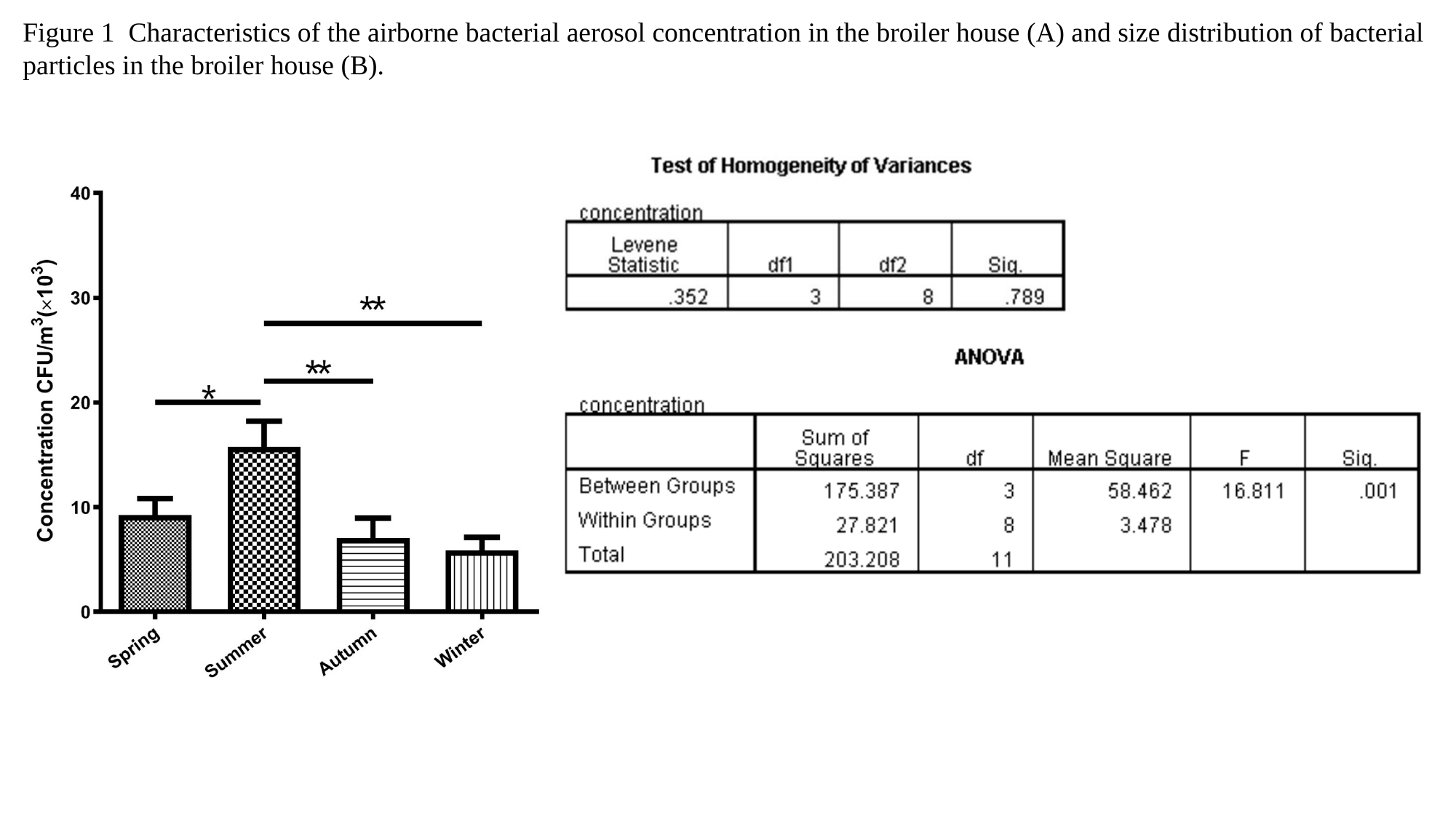

Figure 1 Characteristics of the airborne bacterial aerosol concentration in the broiler house (A) and size distribution of bacterial particles in the broiler house (B).

## Slide 2
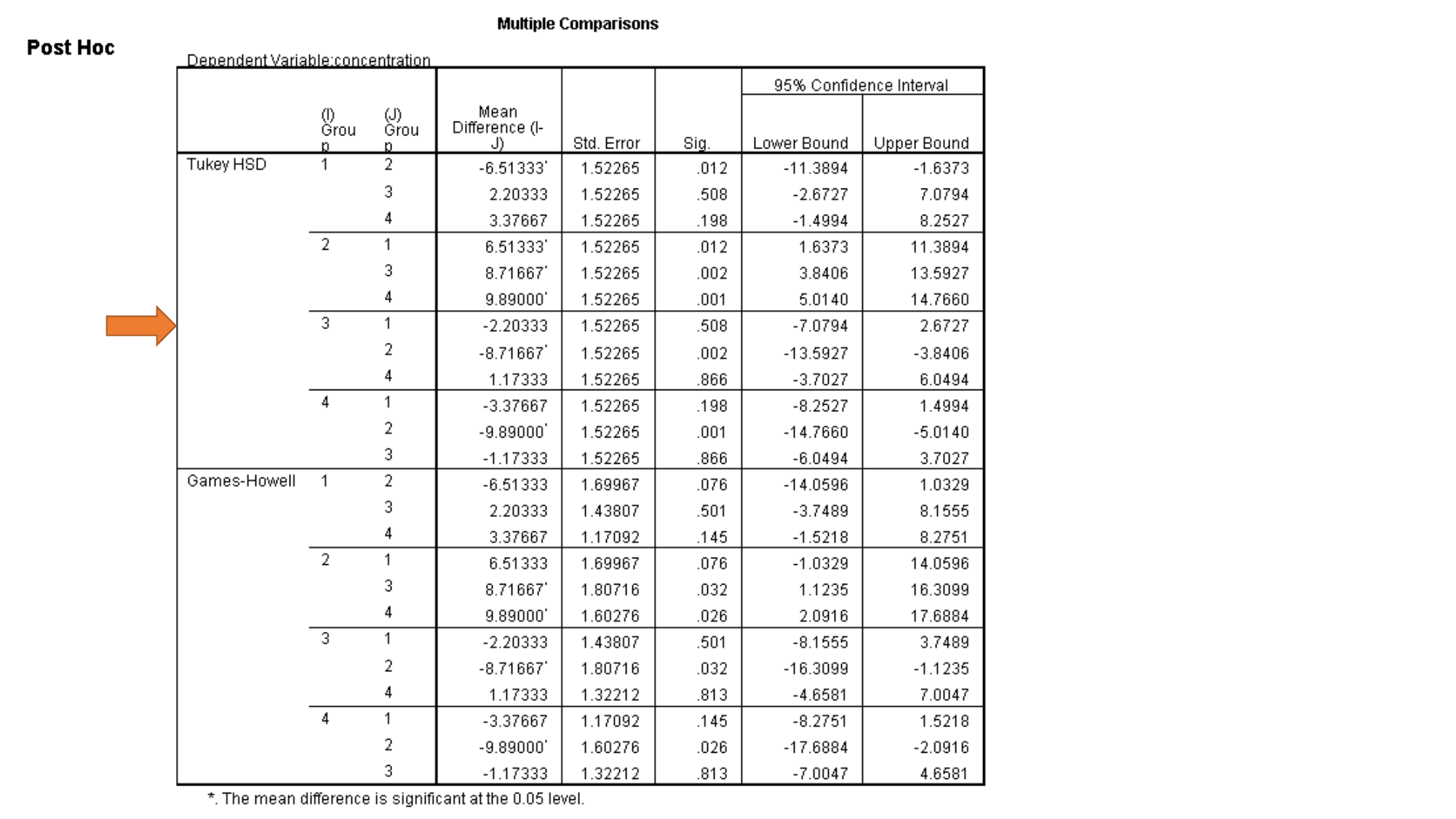

## Slide 3
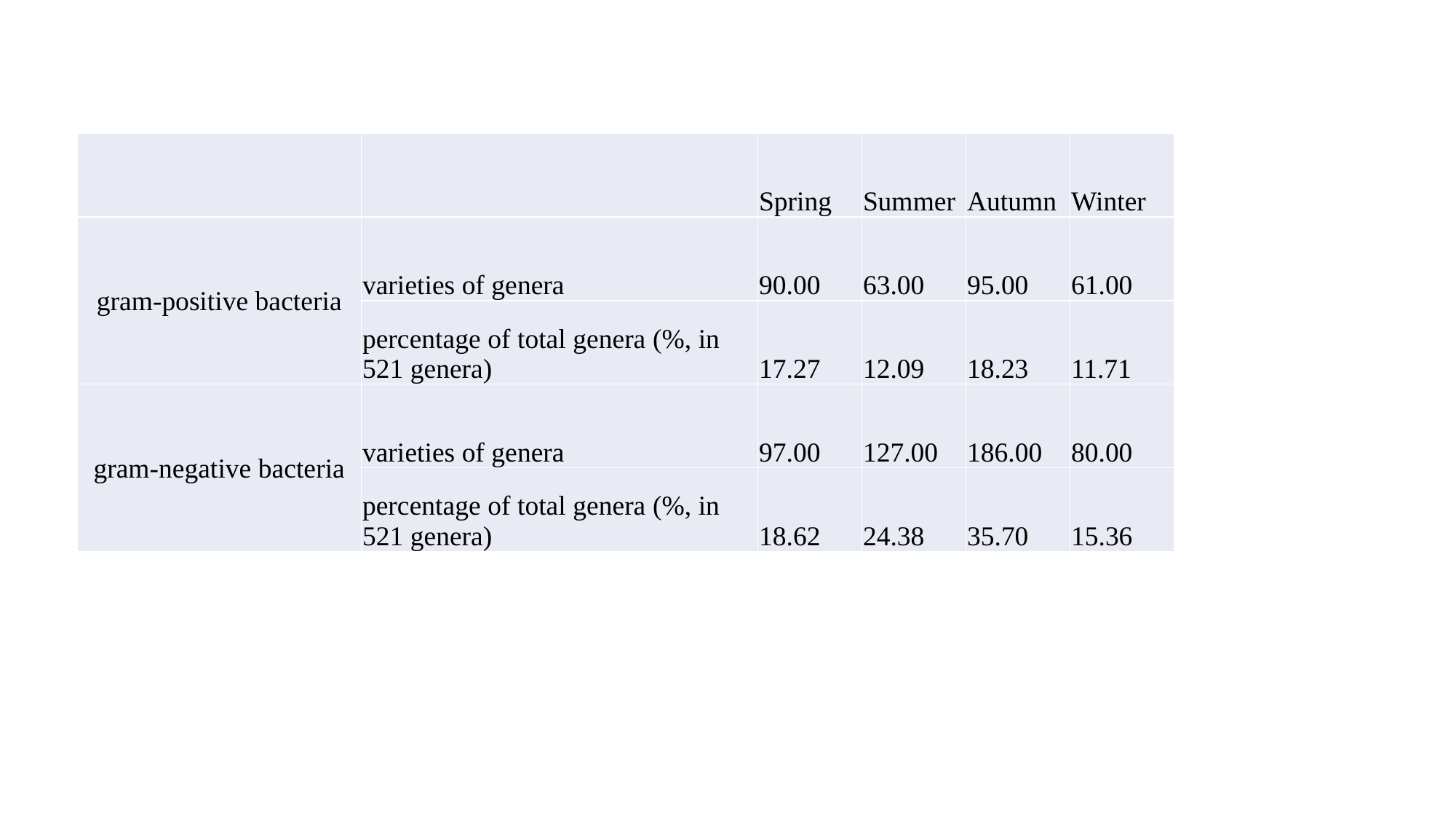

| | | Spring | Summer | Autumn | Winter |
| --- | --- | --- | --- | --- | --- |
| gram-positive bacteria | varieties of genera | 90.00 | 63.00 | 95.00 | 61.00 |
| | percentage of total genera (%, in 521 genera) | 17.27 | 12.09 | 18.23 | 11.71 |
| gram-negative bacteria | varieties of genera | 97.00 | 127.00 | 186.00 | 80.00 |
| | percentage of total genera (%, in 521 genera) | 18.62 | 24.38 | 35.70 | 15.36 |
